# Persistent organic pollutants and non-alcoholic fatty liver disease in morbidly obese patients: a cohort study

**DOI:** 10.1186/s12940-015-0066-z

**Published:** 2015-09-29

**Authors:** Panu Rantakokko, Ville Männistö, Riikka Airaksinen, Jani Koponen, Matti Viluksela, Hannu Kiviranta, Jussi Pihlajamäki

**Affiliations:** 1 National Institute for Health and Welfare, Department of Health Protection, Chemicals and Health Unit, Kuopio, Finland; 2 Department of Medicine, University of Eastern Finland and Kuopio University Hospital, Kuopio, Finland; 3 Department of Environmental Science, University of Eastern Finland, Kuopio, Finland; 4 Institute of Public Health and Clinical Nutrition, Department of Clinical Nutrition, University of Eastern Finland, Kuopio, Finland; 5 Clinical Nutrition and Obesity Center, Kuopio University Hospital, Kuopio, Finland

**Keywords:** Persistent organic pollutants, Perfluorinated alkyl acids, Non-alcoholic fatty liver disease, Obesity, Bariatric surgery

## Abstract

**Background:**

In animal experiments persistent organic pollutants (POPs) cause hepatosteatosis. In epidemiological studies POPs have positive associations with serum markers of nonalcoholic fatty liver disease (NAFLD) and together with obesity synergistic association with insulin resistance. Because insulin resistance and obesity are critical in NAFLD pathogenesis, we investigated the association of serum pollutant levels with liver histology and alanine aminotransferase (ALT) in morbidly obese.

**Methods:**

Liver biopsies were from 161 participants of the Kuopio Obesity Surgery Study (KOBS) who underwent bariatric surgery 2005–2011. Liver histology was categorized as normal, steatosis and non-alcoholic steatohepatitis (NASH). Liver phenotype at baseline and ALT at baseline and 12 months post-surgery were correlated to serum POP concentrations at respective time points. As lipophilic POPs concentrate to smaller fat volume during weight loss, serum levels before and 12 months after bariatric surgery were compared.

**Results:**

Baseline serum concentration of PCB-118, β-HCH and several PFAAs had an inverse association with lobular inflammation possibly due to changes in bile acid metabolism. ALT had negative associations with many POPs at baseline that turned positive at 12 months after major clinical improvements. There was an interaction between some POPs and sex at 12 months, and in stratified data positive associations were observed mainly in females but not in males.

**Conclusions:**

We found a negative association between serum concentrations of PCB-118, β-HCH and several PFAAs with lobular inflammation at baseline. Positive POPs-ATL associations at 12 months among women suggest that increased POP concentrations may decrease the degree of liver recovery.

**Electronic supplementary material:**

The online version of this article (doi:10.1186/s12940-015-0066-z) contains supplementary material, which is available to authorized users.

## Background

Non-alcoholic fatty liver disease (NAFLD) is rapidly becoming the most common cause of liver disease and it is estimated that NAFLD affects 20–30 % of the population in the West [1]. Within the liver, NAFLD can progress to non-alcoholic steatohepatitis (NASH) and liver cirrhosis, and ultimately to liver failure [2]. Furthermore, patients with NAFLD exhibit increased risk of developing type 2 diabetes (T2D), cardiovascular disease (CVD), chronic kidney disease, and certain malignancies [3]. Insulin resistance [4] and obesity [5] are two important elements in the pathogenesis of NAFLD. Both increase the inflow of free fatty acids (FFAs) to the liver from subcutaneous and visceral fat and contribute to “de novo” intrahepatic synthesis of triglycerides [6]. In humans healthy diet [7], weight loss and increased physical activity [8] improve NAFLD. Bariatric surgery has been shown to improve components of the metabolic syndrome, with a dramatic reduction in hepatic steatosis and an improvement in the NAFLD stage [9].

Persistent organic pollutants (POPs) are heterogeneous group of chemicals harmful to humans. In Finland the main exposure routes are the consumption of fatty fish [10] and house dust [11, 12]. Production and use of many POPs has been banned by international treaties. Decreasing time trends of exposure have been observed in the Nordic Countries e.g. for polychlorinated biphenyls (PCBs) and organochlorine pesticides (OCPs) since 1960’s and 1970’s [13], for polybrominated diphenylethers (PBDEs) since 1990’s [14] and for perfluorinated alkyl acids (PFAAs) since 2000’s [15]. Due to their lipophilicity POP are stored in humans primarily in adipose tissue and also in the liver [16], but animal and some human data indicate that dioxin-like compounds are also selectively sequestrated to liver [16, 17]. Contrary to other POPs, PFAAs are not lipophilic and they have been shown to bind to proteins in blood and especially in the liver of animals [18] and to various protein rich tissues also in humans [19].

In rats single high oral dose of certain PCBs [20] and OCPs [21] increases deposition of triglycerides to liver. These effects may also depend on the diet. For example, in rats POP contaminated salmon oil (but not decontaminated) caused hepatosteatosis [22], while in mice POP contaminated whale meat did not [23]. PCBs have also been proposed to act as a “second hit” driving steatosis to NASH in mice exposed to high fat diet [24]. Regarding PFAAs, hepotomegaly is commonly observed as a sign of hepatotoxicity in rodents and nonhuman primates [18].

In humans serum levels of certain lipophilic POPs have been associated with NAFLD related conditions (e.g. T2D and obesity). Regarding T2D, reviews of cross-sectional studies suggest/support a positive association for certain organochlorine POPs, such as *trans*-nonachlor, dichlorodiphenyldichloroethylene (p,p′-DDE), and PCBs [25, 26]. A recent meta-analysis of seven existing prospective studies indicated towards the temporal precedence for hexachlorobenzene (HCB) and total PCBs, but the data was insufficient to establish causality [27]. Regarding obesity, a current review concluded that OC pesticides (especially p,p′-DDE) tend to be positively associated or not associated with obesity, but PCBs have also shown inverse associations in many studies [28]. Two prospective studies have been conducted. One study among young adults observed that p,p′-DDE and PCBs with ≥7 chlorines had inverted U-shaped association with increased body mass index in 18 years follow-up [29]. In another study among elderly adults low-dose exposure to less chlorinated PCBs, p,p′-DDE, and dioxins were associated with the development of abdominal obesity in 5 years follow-up [30]. In addition, POPs and obesity together have a synergistic association with T2D or insulin resistance [31, 32]. Regarding NAFLD itself, an increase in serum PCB levels was associated with alanine aminotransferase (ALT) elevation, a proxy marker of NAFLD among general adult population (NHANES 2003–2004) [33]. To support an interaction with insulin resistance there was a positive association between PFOA and ALT and γ-GT especially in obese and insulin resistant individuals with or without metabolic syndrome in NHANES 1999–2000 and 2003–2004 [34]. Experimental studies suggest that POPs may activate nuclear receptors including aryl hydrocarbon receptor (AhR), pregnane X receptor (PXR) and constitutive androstane receptor (CAR). Through these receptors POPs may induce the regulation of genes involved in the inflammatory pathway, mitochondrial function, lipid oxidation, and lipogenesis, thereby contributing to development of insulin resistance and obesity [22, 28].

Based on the proposed interaction of obesity and insulin resistance with POP exposure, morbidly obese represent a population where the association between POPs and NAFLD is most likely seen if that exists. In addition, because plasma levels of lipophilic POPs increase after diet induced weight loss [35, 36] or bariatric surgery [35, 37] as they concentrate to a smaller adipose tissue volume, the impact of increased POP concentrations after weight loss on the improvement of liver function is of interest. Only one study has explored the metabolic associations of POP levels before and after bariatric surgery. Pre-surgery serum POP levels had significant positive partial correlations (adjusted for age and sex) with liver dysfunction markers (ALT, AST and γ-GT). Using similar test increased post-surgery POP concentrations were associated with a diminished improvement of liver related parameters [37].

Notably, none of the studies investigating the relationship between POPs and liver disease have relied on liver biopsy, the golden standard for the evaluation of NAFLD and NASH [38]. In this study we investigated the association of serum levels of several POPs with detailed liver histology and metabolic parameters in morbidly obese patients. Furthermore, levels before and 1 year after bariatric surgery induced weight loss were compared.

## Methods

### Study population

Altogether 161 subjects (47.6 ± 8.4 years, BMI 44.6 ± 5.7 kg/m^2^) were selected from an ongoing Kuopio Obesity Surgery Study (KOBS) including all subjects undergoing bariatric surgery at the Kuopio University Hospital [39]. Recruitment and surgery of study subjects took place during the years 2005–2011. Selection criteria for the study were according to Finnish Current Care Guidelines that are based on the National Institutes of Health Consensus Development Conference Statement from 1991 [40]. Detailed criteria were 1) BMI >40 kg/m2, 2) BMI 35–40 kg/m2 and comorbidity or its risk factor, such as T2D, hypertension, sleep apnea, osteoarthritis of weight bearing joints or polycystic ovarian syndrome, 3) failure of dietary and drug treatments, and 4) no other contraindication for operation. Over 90 % of the eligible recruited individuals participated. Alcohol consumption of ≤2 doses per day was applied as study inclusion criteria. All participants were on a very low calorie diet for 8 weeks before the surgery (800–1000 kcal/day) during which 5–10 kg weight loss typically occur for all patients. Weight loss in this cohort during the 1 year follow-up was 29.7 ± 11.9 kg (−24.6 %) [39]. Informed consent was obtained from each participant and the study protocol was approved by the Ethics Committee of Northern Savo Hospital District and was in accordance with the Helsinki Declaration.

### Clinical determinations and blood samples

During the week preceding the surgery every participant had one-day visit including an interview on the history of previous diseases, current drug treatment and an evaluation of cardiovascular risk factors. On the morning of surgery before preoperative preparation of patients fasting blood samples were drawn after 12 h of fasting. Fasting blood samples were also collected at 12 months after surgery. The following analyses were carried out at baseline and 1 year after the surgery: Plasma glucose by enzymatic hexokinase photometric assay (Konelab Systems Reagents, Thermo Fischer Scientific, Vantaa, Finland), serum insulin by immunoassay (ADVIA Centaur Insulin IRI, no 02230141, Siemens Medical Solutions Diagnostics, Tarrytown, NY), cholesterol and triglyceride levels from the whole serum and from lipoprotein fractions by automated enzymatic methods (Roche Diagnostics, Mannheim, Germany) and plasma activities of ALT by kinetic method according to the International Federation of Clinical Chemistry (Roche Diagnostics Co).

### Liver biopsies

Liver biopsies were obtained using Trucut needle (Radiplast AB, Uppsala, Sweden) during elective gastric bypass operation (*n* = 161). Histological assessment of liver was performed by one pathologist according to Brunt et al. [41]. Steatosis was graded into 4 categories (<5, 5–33, 33–66 % and >66 %); lobular inflammation into 4 categories (no foci; <2, 2–4 and >4 per 200× field); ballooning was staged from 0 to 2 and fibrosis from 0 to 4. Based on this histological assessment a clear distinct liver phenotype (normal, simple steatosis or NASH) could be determined for 105 of these 161 patients (Table 1).

**Table 1:** Clinical characteristics (mean ± SD) and liver histology by baseline liver phenotype^a^

Liver phenotype at baseline	Normal	Steatosis	NASH	
	*n* = 42	*n* = 28	*n* = 35	p^b^
Sex (male/female) (% males)	15/27 (36)	7/21 (25)	14/21 (40)	0.446
Age (years)	48.2 ± 8.7	47.1 ± 8.7	47.3 ± 8.5	0.910
Weight	128.7 ± 20.4	124.0 ± 15.7	134.4 ± 23.9	0.265
Weight at 12 mo	102.0 ± 20.0	91.1 ± 6.2	98.9 ± 19.8	0.200
BMI (kg/m^2^)	44.0 ± 6.4	43.9 ± 3.9	44.8 ± 6.9	0.824
BMI at 12 mo (kg/m^2^)	35.2 ± 6.4	32.1 ± 2.4	33.3 ± 5.7	0.289
Fasting glucose (mmol/L)	6.0 ± 0.8	6.5 ± 2.2	7.0 ± 2.4	0.287
Fasting glucose at 12 mo (mmol/L)	5.4 ± 0.7	5.4 ± 0.7	5.3 ± 0.5	0.581
Fasting insulin (mU/L)	13.9 ± 6.7	18.7 ± 10.7	39.9 ± 59.1	<0.001
Fasting insulin at 12 mo (mU/L)	8.6 ± 5.2	7.8 ± 3.9	11.3 ± 11.9	0.958
Diabetes (no/yes) (%)^c^	32/10 (24)	18/10 (36)	18/16 (46)	0.105
Diabetes at 12 mo (no/yes) (%)^d^	30/1 (2.4)	17/0 (0)	24/0 (0)	0.511
Cholesterol (mg/l)	4.2 ± 0.9	4.0 ± 0.7	4.7 ± 1.2	0.040
Cholesterol at 12 mo (mg/l)	4.2 ± 0.8	4.2 ± 0.8	4.7 ± 0.9	0.264
Triglycerides (mg/l)	1.5 ± 0.6	1.6 ± 0.7	1.8 ± 0.9	0.076
Triglycerides at 12 mo (mg/l)	1.0 ± 0.4	1.0 ± 0.3	1.2 ± 0.4	0.222
Plasma adiponectin (μg/ml)	3.6 ± 2.0	4.0 ± 2.1	2.8 ± 1.7	0.084
Plasma adiponectin at 12 mo (μg/ml)	13.0 ± 37.2	16.2 ± 43.3	5.4 ± 3.4	0.205
ALT (U/L)	40.0 ± 25.5	43.8 ± 17.9	58.2 ± 31.4	0.042
ALT at 12 mo (U/L)	27.4 ± 12.9	24.9 ± 12.9	27.8 ± 25.3	0.626
Steatosisgrade (n)				<0.001
<5 %	42	0	0	
5–33 %	0	24	14	
33–66 %	0	2	14	
>66 %	0	2	7	
Lobular Inflammation (n)				<0.001
None	42	28	0	
<2 foci per 200*field	0	0	25	
2–4 foci per 200*field	0	0	10	
Liver cell ballooning (n)				<0.001
None	42	28	16	
Few balloon cells (n)	0	0	15	
Many cells/prominent ballooning	0	0	4	
Fibrosis stage (n)				<0.001
None	42	28	4	
Perisinusoidal or periportal	0	0	26	
Perisinusoidal and portan/periportal	0	0	22	
Bridging fibrosis	0	0	12	
Cirrhosis	0	0	1	

^a^Selected clinical characteristics both at baseline and at 12 months and liver histology at baseline, all grouped by liver phenotype at baseline

^b^Kruskal-Wallis test for continuous variables and Chi-square test for categorical variables

^c^Previous doctor diagnosis of diabetes

^d^Fasting glucose ≥7 at 12 months examination

### Chemical analysis of POPs and PFAAs

The method used for the analysis of 17 POPs and 13 PFAAs from serum samples before and 12 months after bariatric surgery has been described previously in detail [42]. POP compounds measured were PCBs 118, 138, 153, 156, 170, and 180, pesticides HCB, β -hexachlorocyclohexane (β-HCH), *trans*-nonachlor, p,p′-DDE, and brominated diphenyl ethers BDE-47, BDE-99, BDE-153 and BDE-209. PFAAs compounds were perfluorohexanesulfonic acid (PFHxS), −octanosulfonic acid (PFOS), perfluorohexanoic acid (PFHxA), −octanoic acid (PFOA), −nonanoic acid (PFNA), −decanoic acid (PFDA) and -undecanoic acid (PFUnA). Also some other POPs and PFAAs were measured, but for those more than 70 % of samples had concentrations less than the limit of quantification (LOQ) and are not treated further. To achieve better sensitivity especially for higher brominated PBDEs, the analysis of PBDEs was performed with an Agilent 6890 gas chromatograph equipped with a short column (J&W Scientific DB-5MS-UI: 6 m, ID 0.18 mm, 0.18 μm) connected to Waters Autospec Ultima high resolution mass spectrometer (GC-HRMS). All concentrations of POPs are expressed as ng/g of serum lipid whereas concentrations of PFAAs are expressed as ng/ml of serum.

For POPs 2 blanks and 2 control samples (NIST SRM1958) were included in each batch of samples (*n* = 9). Measured concentrations of POPs in SRM1958 were 80–105 % of the certified/reference concentrations, except for BDE-209 for which it was 72 %. Co-efficient of variation (CV-%) from SRM 1958 (*n* = 18) was <3.6 % for all compounds except for BDE-209 (16.7 %). For PFAAs one blank and one in-house control serum sample were included in each batch of samples (*n* = 4). PFAAs control sample was prepared by spiking typical serum sample with 5.0 ng/ml of each PFAA analysed. CV-% from in-house control serum (*n* = 4) ranged from 3.6 to 12.6 % depending on the compound. During the POP/PFAAs analysis the laboratory participated to AMAP interlaboratory comparison rounds one and two of 2013 (Ring Test for Persistent Organic Pollutants in human serum, National Institute of Public Health, Quebec, Canada). For POPs laboratory results varied from 83 to 132 % and for PFAAs from 79 to 128 % of the assigned values depending on the compound. LOQs were 2–15 pg/ml for POP and 0.08–0.18 ng/ml for PFAAs.

### Statistics

Kruskal-Wallis test for continuous variables and Chi-square test for categorical variables were used to study the difference between clinical characteristics of patients at baseline and at 12 months grouped by liver histology at baseline. Percent changes in the concentrations of each PFAA and POP compound from baseline to 12 months were calculated separately for each individual study subject who had serum sample available at both time points. Percent changes were calculated using concentrations in the original scale as 100*([conc. at 12 mo]-[conc. at baseline]/[conc. at baseline]). For each compound mean, median, 5th and 95th percentiles were calculated from percent changes in individual study subjects. Differences in the serum POP and PFAAs concentrations before and after bariatric surgery were tested by Wilcoxon signed-rank test. These percent changes in the serum contaminant concentrations from baseline to 12 months are denoted with ΔPFAAs or ΔPOPs.

Two different models were used to study the associations between liver histology at baseline and log-transformed concentrations of POPs and PFAAs by multinomial logistic regression. The first one was adjusted only for age and the second one was adjusted for age, BMI, sex and fasting insulin (PFAAs also for serum lipids). Stratification by sex was not performed due to small number of cases in certain classes of liver histology. BMI and serum insulin were added in the models because they are central etiological factors in NAFLD development.

Associations between log-transformed serum ALT concentrations and log-transformed serum POPs (lipid based) and PFAA concentrations (volume based) at baseline and at 12 months were studied by linear regression. At baseline models adjusted for age and models adjusted for age, sex, BMI and fasting insulin were run. Similar adjustments were performed at 12 months, but BMI was replaced with weight change in kilos as weight loss may be a better predictor of the chemical blood concentrations than BMI at 12 months. Interaction between sex and POPs was tested in separate models at baseline and at 12 months by including sex*POPs interaction term to fully adjusted models. Finally, fully adjusted models between ALT and POPs stratified by sex were also run.

To test the possibility of inflammation related pre-surgery accumulation of POPs to liver and their post-surgery release to serum, ΔPOP and ΔPFAAs were calculated in different liver inflammation status groups. Same tests with liver phenotype as grouping variable were also performed. Differences in the percent changes between groups were tested by Kruskal-Wallis test.

In all statistical analysis results < LOQ were treated as LOQ/2. In statistical analysis *p*-values <0.05 were considered significant. All statistical tests were performed with IBM SPSS Statistics 21, Armonk, New York, USA.

## Results

### Clinical characteristics

Of the clinical parameters, the difference between study groups (normal liver, simple steatosis and NASH) in fasting insulin level at baseline was highly significant mainly due to high insulin level in the NASH group (Table 1). Significant differences were also observed for fasting cholesterol and ALT at baseline.

### Change in the levels of POPs and PFAAs from baseline to 12 months

Changes in the serum PFAAs concentrations in response to surgery (ΔPFAAs) were generally very small (for median range −6.5–4.3 %) even though some changes were significant in the Wilcoxon signed-rank test (Table 2). The median of ΔPOPs for chlorinated lipid soluble POP were significant (+39–+77 %) as expected, and for many compounds 95th percentiles of ΔPOPs were around +200 %. However, the behaviour of PBDEs was more variable: for BDE47 and BDE209 median changes in serum concentrations were small (12 and 11 %), but for BDE153 all changes (mean, median, percentiles) were similar to those for chlorinated POPs (Table 2).

**Table 2:** Concentrations of PFAAs, OCPs, PCBs and PBDEs before and 12 months after bariatric surgery

	Before surgery			12 months after surgery			Change^a^		
		Mean	Median (5th; 95th persentile)		Mean	Median (5th, 95th pers.)	Mean	Median (5th; 95th persentile.)	p-value*
Compound	n^c^	ng/ml	ng/ml	n^c^	ng/ml	ng/ml	%	%	
PFHxA	161	0.16	0.03 (0.03; 0.59)	118	0.16	0.03 (0.03, 0.56)	150^b^	0.00 (-92; 1108)	0.67
PFOA	161	2.63	2.56 (1.04; 4.66)	118	2.60	2.50 (1.00, 4.80)	0.1	-4.8 (-30; 33)	0.017
PFNA	161	0.96	0.83 (0.30; 2.19)	118	0.97	0.84 (0.29, 2.55)	-1.2	-6.5 (-38; 46)	0.029
PFDA	161	0.27	0.23 (0.08; 0.60)	117	0.27	0.24 (0.08, 0.72)	4.4	-5.6 (-52; 92)	0.258
PFUnA	161	0.19	0.15 (0.02; 0.50)	118	0.18	0.13 (0.02, 0.54)	11	-3.3 (-77; 142)	0.042
PFHxS	161	1.42	1.18 (0.54; 2.90)	118	1.46	1.35 (0.50, 2.88)	9.9	4.3 (-50; 89)	0.332
PFOS	161	3.91	3.2 (0.89; 10.3)	118	3.91	3.32 (0.98, 8.76)	1.1	-3.4 (-32; 50)	0.038
sumPFCA	161	4.20	4.07 (1.8; 7.52)	118	4.17	3.94 (1.64, 7.77)	-1.0	-6.1 (-29; 33)	0.003
sumPFSA	161	5.33	4.49 (1.75; 12.3)	118	5.37	4.82 (1.93, 11.3)	1.2	-2.1 (-31; 45)	0.222
		Mean	Median (5th; 95th pers.)		Mean	Median	Mean	Median (5th; 95th pers.)	p-value*
Compound	n^c^	ng/g lipid	ng/g lipid	n^c^	ng/g lipid	ng/g lipid	%	%	
HCB	149	12.8	10.9 (5.24; 23.3)	95	24.1	18.3 (7.31, 56.9)	113	77 (-12; 330)	<0.001
BetaHCH	149	17.1	12.7 (2.60; 28.6)	95	19.2	17.6 (3.79, 41.3)	49	39 (-19; 147)	<0.001
Transnonachlor	149	9.30	7.26 (1.59; 24.8)	95	14.5	10.9 (1.92, 41.6)	73	58 (-1.5; 190)	<0.001
p,p′-DDE	149	151	100 (16.8; 504)	95	229	157 (23.3, 688)	73	67 (-2.0; 182)	<0.001
PCB-118	149	13.3	10.0 (3.09; 33.3)	95	21.3	18.4 (4.82, 52.8)	68	62 (-8.6; 182)	<0.001
PCB-153	149	75.4	62.8 (19.4; 193)	95	125	108 (28.4, 314)	73	61 (-0.10; 188)	<0.001
PCB-138	149	39.8	32.2 (10.0; 103)	95	64.5	55.3 (14.0, 181)	71	65 (6.4; 176)	<0.001
PCB-156	149	3.98	3.40 (0.91; 9.88)	95	6.64	6.15 (1.11, 15.5)	84	65 (-8.7; 254)	<0.001
PCB-180	149	42.0	35.8 (11.2; 109)	95	69.2	62.0 (13.9, 164)	74	60 (-19; 196)	<0.001
PCB-170	149	18.1	15.5 (4.74; 46.2)	95	29.7	28.0 (6.38, 65.4)	70	59 (-3.5; 182)	<0.001
BDE-47	149	3.47	1.99 (1.11; 7.01)	95	4.48	2.17 (1.33, 12.2)	31	12 (-40; 183)	0.001
BDE-153	149	1.32	0.92 (0.31; 3.54)	95	2.40	1.53 (0.33, 8.82)	80	61 (-27; 241)	<0.001
BDE-209	149	11.8	4.79 (1.60; 48.5)	95	15.4	5.02 (2.07, 62.7)	213	11 (-88; 1132)	0.436
sumPCB	149	193	163 (50.8; 482)	95	316	271 (72.2, 786)	72	62 (-3.6; 186)	<0.001
sumBDE	149	16.6	8.94 (3.71; 59.5)	95	22.3	11.8 (4.83, 75.6)	115	21 (-79; 633)	0.036

*p-value from Wilcoxon signed-rank test

^a^Mean, median, 5th and 95th persentile of serum PFAAs and POP concentration changes from baseline to 12 months were calculated from changes in individual study subjects who had serum sample available at both time points (same n as in the 12 months after surgery column)

^b^A few extreme values rise the mean of percent change, but median remains 0 as substantial number of results were < LOQ both before and after surgery

^c^For POPs n is smaller than for PFAAs both at baseline and at 12 months because for part of the study subjects lipid determination was not done at either time point

### POPs, PFAAs and liver histology at baseline

Associations between liver histology at baseline and log-transformed concentrations of β-HCH and PCB118 are shown in Table 3 (model adjusted for age, BMI, sex and fasting insulin). β-HCH had significant negative association with lobular inflammation (2–4 foci per 200x field, *p* = 0.022). For dioxin-like PCB-118 there was a negative association with NASH (*p* = 0.038), lobular inflammation (<2 foci per 200x field, *p* = 0.027, and 2–4 foci per 200x field, *p* = 0.005), liver cell ballooning (few balloon cells *p* = 0.036) and steatosis grade (33–66 %, *p* = 0.048). In addition, serum concentrations of both β-HCH and PCB-118 generally decreased with impairment of all liver histology measures (Table 3). Associations for β-HCH and PCB-118 in the models adjusted only for age were mainly similar to those in the fully adjusted model. However, in fully adjusted model association of PCB-118 with diagnosis and steatosisgrade was significant and the precision of the effect estimates was increased as compared to models adjusted only for age (Table 3 and Additional file 1: Table S1). Associations for other POPs were also mainly negative but non-significant and are not tabulated.

**Table 3:** Associations of liver histology with β-HCH and PCB118 at baseline^a,b^

		β-HCH			PCB-118		
	n	Median (ng/g lipid)	OR (95 % CI)	*p*-value	Median (ng/g lipid)	OR (95 % CI)	*p*-value
Diagnosis							
Normal	38	13.4	Ref		15.2	Ref	
Steatosis	28	12.8	3.03 (0.46; 20.10)	0.250	9.42	0.49 (0.07; 3.46)	0.473
NASH	31	11.9	0.33 (0.03; 3.73)	0.373	10.1	0.10 (0.01; 0.88)	0.038
Lobular Inflammation							
None	92	13.2	Ref		11.1	Ref	
<2 foci per 200x field	47	12.5	0.55 (0.11; 2.77)	0.468	9.14	0.16 (0.03; 0.81)	0.027
2–4 foci per 200x field	10	8.93	0.02 (<0.01; 0.59)	0.022	5.55	0.01 (<0.01; 0.26)	0.005
Liver cell ballooning							
None	109	13.3	Ref		11.0	Ref	
Few balloon cells	36	12.0	0.16 (0.02; 1.09)	0.061	9.23	0.17 (0.03; 0.89)	0.036
Many cells/prominent ballooning	4	8.65	0.35 (0.01; 23.27)	0.623	7.31	0.33 (0.00; 22.9)	0.606
Steatosisgrade							
<5 %	50	13.1	Ref		12.6	Ref	
5–33 %	59	13.5	1.09 (0.24; 4.83)	0.914	9.83	0.30 (0.06; 1.49)	0.141
33–66 %	23	11.9	0.19 (0.02; 2.15)	0.180	9.15	0.11 (0.01; 0.98)	0.048
>66 %	17	10.4	0.20 (0.02; 2.51)	0.213	9.14	0.14 (0.01; 1.44)	0.098

^a^Results reported only for β-HCH and PCB118 because other POPs had no significant associations

^b^Concentrations of β-HCH and PCB-118 (ng/g lipids) were log-transformed for the multinomial logistic regression analysis that was adjusted for age, BMI, sex and fasting insulin

Regarding PFAAs, in the fully adjusted model lobular inflammation (2–4 foci per 200x field) had significant negative associations at baseline with PFOA (*p* = 0.027), PFNA (*p* = 0.019), PFDA (*p* = 0.037), PFHxS (*p* = 0.018) and sum of PFCA (*p* = 0.015) (Table 4). Respective associations in the models adjusted only for age were all non-significant and the precision of the effect estimates was decreased (Additional file 2: Table S2). No significant associations with other liver histology parameters at baseline were observed.

**Table 4:** Associations of lobular inflammation status with PFAAs at baseline^a^

	Lobular Inflammation status							
	None (*n* = 96)		<2 foci per 200x field (*n* = 53)			2–4 foci per 200x field (*n* = 11)		
Compound^a^	Median (ng/ml)		Median (ng/ml)	OR (95 % CI)	*p*-value	Median (ng/ml)	OR (95 % CI)	*p*-value
PFHxA	0.060	Ref	0.025	0.80 (0.38; 1.66)	0.547	0.083	0.77 (0.19; 3.03)	0.707
PFOA	2.61	Ref	2.61	0.71 (0.10; 5.18)	0.734	1.823	0.02 (<0.01; 0.66)	0.027
PFNA	0.84	Ref	0.86	0.29 (0.05; 1.61)	0.157	0.64	0.02 (<0.01; 0.53)	0.019
PFDA	0.23	Ref	0.29	1.24 (0.26; 5.90)	0.787	0.15	0.05 (<0.01; 0.83)	0.037
PFUnA	0.15	Ref	0.14	0.73 (0.29; 1.85)	0.511	0.12	0.23 (0.05; 1.15)	0.073
PFHxS	1.21	Ref	1.12	0.25 (0.03; 1.82)	0.170	0.89	0.02 (<0.01; 0.53)	0.018
PFOS	3.30	Ref	3.10	0.52 (0.13; 2.09)	0.353	2.35	0.14 (0.01; 1.66)	0.119
sumPFCA	3.96	Ref	4.04	0.49 (0.05; 4.47)	0.527	3.29	0.01 (<0.01; 0.39)	0.015
sumPFSA	4.82	Ref	4.84	0.42 (0.07; 2.57)	0.349	4.58	0.05 (0.00; 1.50)	0.083

^**a**^Concentrations of PFAAs (ng/ml) were log-transformed for the multinomial logistic regression analysis that was adjusted for age, sex, BMI, serum lipids and fasting insulin

### POPs, PFAAs and serum ALT at baseline and at 12 months

Serum ALT was measured both at baseline and at 12 months. In the fully adjusted models at baseline all chlorinated POPs had negative association with ALT that reached statistical significance for β-HCH and PCBs 153, 180 and 170. In the fully adjusted models at 12 months these associations turned positive and were significant for PCB-118, PCB-156 and BDE-153 (Table 5). In the models adjusted only for age associations were much weaker at baseline, but only slightly weaker at 12 months (Additional file 3: Table S3). At 12 months most POPs had significant or nearly significant interaction with sex (Table 5). In the analysis stratified by sex significant or nearly significant associations between POPs and ALT were most consistently observed among women at 12 months (Additional file 4: Table S4).

**Table 5:** Associations between ALT and POPs at baseline and at 12 months

	Baseline (*n* = 115)^a^			12 months (*n* = 92)^b^		
Compound	B (95 % CI)	p-value	p-interaction^c^	B (95 % CI)	p-value	p-interaction^c^
HCB	-0.20 (-0.40,0.00)	0.055	0.220	0.09 (-0.09,0.28)	0.309	0.256
β-HCH	-0.17 (-0.33,-0.01)	0.035	0.070	0.18 (-0.02,0.39)	0.075	0.017
Trans-nonachlor	-0.11 (-0.26,0.04)	0.133	0.310	0.15 (0.00,0.30)	0.055	0.035
p,p′-DDE	-0.05 (-0.16,0.07)	0.446	0.759	0.11 (-0.01,0.24)	0.076	0.702
PCB - 118	-0.09 (-0.25,0.07)	0.275	0.414	0.19 (0.02,0.36)	0.032	0.074
PCB - 153	-0.21 (-0.42,-0.01)	0.043	0.567	0.18 (-0.02,0.38)	0.083	0.080
PCB - 138	-0.17 (-0.34,0.00)	0.052	0.576	0.14 (-0.05,0.33)	0.138	0.086
PCB - 156	-0.19 (-0.39,0.01)	0.065	0.683	0.19 (0.00,0.38)	0.047	0.053
PCB - 180	-0.30 (-0.56,-0.04)	0.023	0.832	0.20 (-0.01,0.41)	0.064	0.081
PCB - 170	-0.33 (-0.60,-0.06)	0.019	0.762	0.19 (-0.03,0.42)	0.093	0.093
BDE - 47	0.08 (-0.06,0.22)	0.260	0.866	0.07 (-0.07,0.20)	0.326	0.051
BDE - 153	0.11 (-0.02,0.23)	0.085	0.594	0.15 (0.04,0.26)	0.009	0.508
BDE - 209	-0.01 (-0.10,0.08)	0.801	0.705	0.02 (-0.08,0.11)	0.738	0.210
SumPCBs	-0.24 (-0.46,-0.02)	0.033	0.592	0.19 (-0.02,0.39)	0.078	0.071
SumBDEs	0.03 (-0.09,0.14)	0.662	0.580	0.07 (-0.05,0.19)	0.248	0.028

^a^Concentrations of POPs (ng/g lipids) and ALT were log-transformed for the linear regression analysis at baseline that was adjusted for age, sex, BMI and fasting insulin

^b^Concentrations of POPs (ng/g lipids) and ALT were log-transformed for the linear regression analysis at 12 months that was adjusted for age, sex, weight change and fasting insulin

^c^p-value for interaction between each POP and sex was tested in a model where the POP*sex interaction term was added as a covariate in addition to other covariates used at baseline and at 12 months

Of the PFAAs, only PFHxA had significant positive association (*p* = 0.011) with ALT at 12 months in the model adjusted for age, fasting insulin and weight change (results not shown).

### Change in the levels of POPs and PFAAs and baseline liver inflammation

Only BDE-153 had significant difference in the percent change of concentrations from baseline to 12 months between different baseline liver phenotype groups (*p* = 0.009).

## Discussion

This is the first study where serum concentrations of POPs and PFAAs have been explored in relation to liver phenotype (normal, simple steatosis, NASH) as determined by liver biopsy. Participants also had a follow-up visit 12 months after the bariatric surgery where POP, PFAAs and clinical parameters were re-determined following substantial weight loss. This study had two main findings. First, there was an inverse association in the serum concentration of dioxin-like PCB-118 with lobular inflammation at baseline. β-HCH and several PFAAs had similar associations, but less clear (Tables 3 and 4). Second, ALT, as a marker of liver disease, had negative association with POPs at baseline that turned positive at 12 months after major improvements in the liver function tests. For most POPs these associations were significant or nearly significant at both time points (Table 5). In addition, there was an interaction between some POPs and sex at 12 months (Table 5), and stratified data revealed positive associations between ALT and POPs in females but not in males (Additional file 4: Table S4).

For POPs and PFAAs increased accumulation to liver or increased excretion with disease progression could be proposed as explanations to inverse associations. From animal and human post mortem studies accumulation of many dioxin-like compounds to liver is known to take place by dose-dependent induction of CYP1A2 protein capable of hepatic sequestration of these compounds. However, as PCB-118 and β-HCH did not correlate with CYP1A2 expression and did not accumulate to liver in these studies [16, 43], and as decreased CYP1A2 protein expression levels were observed in liver microsomes of patients with NASH [44], possible hepatic accumulation should take place by other mechanisms. For PFAAs a rat study showed that multiple proteins in the liver are capable for specific binding to PFOA, but those proteins were not specified [45]. Unfortunately, from the very limited amount of liver tissue available it was not possible to analyse the level of POPs and PFAAs and compare them to the levels in serum.

Regarding excretion, the presence of POPs in human bile from autopsy samples with significant relationship to concentrations in blood, adipose fat and liver indicates biliary excretion of POPs [46, 47]. NASH patients generally have increased levels of bile acids in both plasma and liver tissue [48, 49] and an increase of serum bile acids in NASH as compared to less severe stages of NAFLD has been found in obese patients undergoing bariatric surgery [50]. In addition, normally a substantial portion of POPs excreted in bile is reabsorbed by the intestine after deconjugation of bile salts by intestinal microflora [51], but the possibility of impaired enterohepatic circulation of bile acids in those with NASH has been suggested [52]. Thus, increase in biliary elimination with NAFLD progression could explain the generally inverse associations of serum POPs with NASH and liver inflammation that reached statistical significance for PCB-118 and β-HCH. Contrary to our results, in former lindane manufacturers liver disease (elevated AST, ALT or γ-GT) was positively correlated with longer β-HCH elimination half-life from blood [53]. However, without information of NASH status, the relevance of this compared to our findings is unsure.

For PFAAs, the urine was concluded to be the major elimination route for short chain perfluorinated carboxylic acids (PFCAs) (C ≤8), but for longer PFCAs, PFOS and PFHxS other non-specified routes of excretion likely contributed to overall elimination [54]. Thus, it cannot be deduced whether increased biliary excretion with NASH might explain the inverse associations observed between some PFAAs and lobular inflammation. However, our results at baseline are generally opposite to epidemiological and occupational studies where a slight positive association between PFOA and liver enzymes (ALT and γ-GT) in obese subjects was observed [34, 55].

In part of the models the number of covariates included increased the significance of associations and improved the precision of effect estimates. Regarding the association of PCB-118 with diagnosis and steatosisgrade (Table 3 and Additional file 1: Table S1) serum insulin level was the key covariate. In addition to a significant difference in the insulin levels between different diagnosis groups at baseline (Table 1), insulin also correlated with liver inflammation (R^2^ = 0.207, *p* = 0.008) and steatosisgrade (R^2^ = 0.217, *p* = 0.006). Similarly, for PFAAs and inflammation (Table 4 and Additional file 2: Table S2), the most important covariate was serum lipids followed by serum insulin, sex and BMI. Here, serum lipids correlated with inflammation (R^2^ = 0.269, *p* = 0.001). As insulin, and possibly also lipids may be a confounders in the models, related to POPs [32], PFAAs [34] and NAFLD [4], they can be expected to have an impact on the effect estimates but less so on their precision. However, the mechanism by which e.g. impaired insulin sensitivity would change the distribution of POPs in this population of morbidly obese is not known.

Change of associations between POPs and ALT from negative at baseline to positive at 12 months and their sex specificity was very interesting (Table 5). Positive POP-ALT associations among women at 12 months (Additional file 4: Table S4) are in line with other metabolic outcomes. For example, in a 25-year follow-up study higher baseline PCB serum levels were associated with increased incidence of T2D in women, but not in men [56]. However, because effect estimates were similar among men for many of the POPs, it is also possible that smaller number of men may in part be the reason for less significant results (Additional file 4: Table S4). The French bariatric surgery cohort observed a positive association between a diminished improvement of liver markers and increased plasma POP concentrations after surgery, but no stratification by sex was performed. This was interpreted as the beneficial effects of weight loss possibly being slowed down or decreased by higher (hepatotoxic) POP levels [37]. Our results among females at 12 months can be interpreted similarly. As a whole, at 12 months increase in POP levels plus improvement in the general clinical situation both increase the likelihood of POP-related ALT signal to be observed if such exists. Also, at 12 months significant clinical differences between baseline groups of liver phenotype had vanished (Table 1) making the study material more homogenous and similar to the general population (NHANES) that showed positive associations between liver enzymes and POPs [33].

The mean/median baseline concentrations of most POPs (especially PCBs) in this study were very similar to those reported recently in other POPs-bariatric surgery cohorts from France [37], Belgium [57], Canada [35] and South-Korea [36]. Although there was a significant increase in POP levels in response obesity surgery for most compounds, but mean ΔPOPs in our study were slightly [37] or much lower [35, 57] than those observed in previous studies 12 months after bariatric surgery. This can in part be explained by the differences in the BMI decreases (ΔBMIs) attained by surgery between studies. Hue et al. reported that ΔPOPs is not monotonic for organochlorine POPs, but shows a much faster rate of increase for ΔBMI >14 kg/m^2^. In his study morbidly obese subjects who underwent bariatric surgery had at 12 months ΔBMI 16–26 kg/m^2^ (*n* = 8) and a mean ΔPOPs of 390 % for the sum of plasma organochlorine compounds [35]. In our total data ΔBMIs and ΔPOPs were more modest; the 95th percentiles were 17.5 kg/m^2^ for ΔBMI and depending on the compound 150-330 % for ΔPOPs, respectively (Table 2).

To our knowledge no previous study has explored changes in serum PFAAs concentrations following diet or bariatric surgery induced weight loss. The known tendency of PFAAs to bind with proteins in blood and especially in liver [18] rather than in fat was indirectly seen in the results of this study, where only minor (<7 % for all studied PFAAs) changes in the median serum concentrations following weight loss were observed. Median baseline PFAAs concentrations in the current study were lower than those reported e.g. in the general US [34] and Norway [58] adult population.

Obesity management prior to recruitment was similar for all patients, i.e. they had non-satisfactory response to conservative treatment given according to Finnish Current Care Guidelines (e.g. counselling on diet and physical exercise). Possible weight losses during pre-recruitment period and pre-surgery low calorie diet period were not recorded. It can be speculated that patients with a better compliance to their physician recommendation prior to surgery would more likely have lost weight which could have led to both an increase in POP levels in blood and an improvement in liver lobular inflammation. Our results at baseline reflect cross-sectional situation on the day of surgery.

This study has several limitations. First, the number of subjects was limited in each class liver phenotype class. However, clear characterization in different liver phenotypes gave a possibility to compare normal liver to steatosis and NASH. Second, there is a risk of false findings due to multiple testing. Third, analysis of POPs from liver biopsies would have been extremely valuable additional information, but it was not possible due to very limited amount of tissue available. Fourth, follow-up liver biopsies at 12 months would also have been valuable, but receiving they are hard to justify ethically. Fifth, study was cross-sectional in nature and the causality for the main findings cannot be established.

## Conclusions

Using a special cohort of obese individuals with high risk of NASH that underwent bariatric surgery we found a negative association between serum concentrations of PCB-118, β-HCH and several PFAAs with lobular inflammation at baseline. Reason for this is unclear, but increase in biliary elimination with NASH could be proposed as one explanation especially for POPs. ALT had significant or nearly significant negative associations with most POPs at baseline that turned positive at 12 months after major improvements in clinical status. There was an interaction between some POPs and sex at 12 months, and in stratified data positive associations were observed mainly in females but not in males. Our results at 12 months agree with the associations observed previously between POPs and increased ALT, used as surrogate for liver disease.

## Additional files

Additional file 1: Table S1. Associations of liver histology with β-HCH and PCB118 at baseline. (PDF 80 kb)
Additional file 2: Table S2. Associations of lobular inflammation status with PFAAs at baseline. (PDF 95 kb)
Additional file 3: Table S3. Associations between ALT and POPs at baseline and at 12 months. (PDF 90 kb)
Additional file 4: Table S4. Associations of ALT with POPs at baseline and at 12 months stratified by sex. (PDF 165 kb)

## Abbreviations

BMI: Body mass index
CV: Co-efficient of variation
CVD: Cardiovascular disease
KOBS: Kuopio Obesity Surgery Study
ΔPFAAs: Percent change in PFAAs concentrations from baseline to 12 months
ΔPOPs: Percent change in POPs concentrations from baseline to 12 months
LOQ: Limit of quantification
NAFLD: Non-alcoholic fatty liver disease
NASH: Non-alcoholic steatohepatitis
OCPs: Organochlorine pesticides
OGTT: Oral glucose tolerance test
OR: Odds ratio
PBDEs: Polybrominated diphenylethers
PFAAs: Perfluorinated alkyl acids
POPs: Persistent organic pollutants
T2D: Type 2 diabetes
