# Supplementary material for: Persistent organic pollutants and non-alcoholic fatty liver disease in morbidly obese patients: a cohort study
Source: Environ Health. 2015 Sep 29;14:79. doi: 10.1186/s12940-015-0066-z (PMC4588245; doi:10.1186/s12940-015-0066-z)
Supplement: Additional file 1: Table S1. — Associations of liver histology with β-HCH and PCB118 at baseline. (PDF 80 kb) [file 12940_2015_66_MOESM1_ESM.pdf]

Table S1. Associations of liver histology with β-HCH and PCB118 at baseline.[a, b]

	n	β-HCH Median (ng/g lipid)	OR (95% CI)	p-value	PCB118 Median (ng/g lipid)	OR (95% CI)	p-value
Diagnosis							
Normal	38	13.4	Ref		15.2	Ref	
Steatosis	28	12.8	3.16 (0.47; 21.4)	0.239	9.42	0.62 (0.09; 4.16)	0.622
NASH	31	11.9	0.42 (0.05; 3.74)	0.436	10.1	0.21 (0.03; 1.39)	0.105
Lobular Inflammation							
None	92	13.2	Ref		11.1	Ref	
<2 foci per 200*field	47	12.5	0.51 (0.11; 2.38)	0.393	9.14	0.21 (0.05; 0.97)	0.046
2-4 foci per 200*field	10	8.93	0.02 (<0.01; 0.53)	0.018	5.55	0.02 (<0.01; 0.41)	0.01
Liver cell ballooning							
None	109	13.3	Ref		11.0	Ref	
Few balloon cells	36	12.0	0.17 (0.03; 1.07)	0.059	9.23	0.19 (0.04; 0.91)	0.038
Many cells/prominent ballooning	4	8.65	0.53 (0.01; 37.3)	0.770	7.31	0.53 (0.01; 31.4)	0.758
Steatosisgrade							
<5%	50	13.1	Ref		12.6	Ref	
5-33%	59	13.5	1.02 (0.24; 4.42)	0.974	9.83	0.36 (0.08; 1.71)	0.201
33-66%	23	11.9	0.18 (0.02; 1.87)	0.151	9.15	0.16 (0.02; 1.25)	0.081
>66%	17	10.4	0.20 (0.02; 2.41)	0.204	9.14	0.20 (0.02; 1.99)	0.171

[a] Results reported only for β-HCH and PCB118 for comparison with Table 3.

[b] Concentrations of β-HCH and PCB118 (ng/g lipids) were log-transformed for the multinomial logistic regression analysis that was adjusted only for age.
