# Supplementary material for: Persistent organic pollutants and non-alcoholic fatty liver disease in morbidly obese patients: a cohort study
Source: Environ Health. 2015 Sep 29;14:79. doi: 10.1186/s12940-015-0066-z (PMC4588245; doi:10.1186/s12940-015-0066-z)
Supplement: Additional file 2: Table S2. — Associations of lobular inflammation status with PFAAs at baseline. (PDF 95 kb) [file 12940_2015_66_MOESM2_ESM.pdf]

Table S2. Associations of lobular inflammation status with PFAAs at baseline[a].

	Lobular Inflammation status							
	None (n = 96)		<2 foci per 200*field (n=53)			2-4 foci per 200*field (n=11)		
Compound	Median (ng/ml)		Median (ng/ml)	OR (95% CI)	p-value	Median (ng/ml)	OR (95% CI)	p-value
PFHxA	0.06	Ref	0.025	0.63 (0.33; 1.22)	0.169	0.083	0.97 (0.30; 3.16)	0.959
PFOA	2.61	Ref	2.61	0.76 (0.13; 4.46)	0.762	1.823	0.06 (0.00; 1.20)	0.066
PFNA	0.84	Ref	0.86	0.96 (0.23; 3.97)	0.952	0.64	0.15 (0.01; 2.16)	0.163
PFDA	0.23	Ref	0.29	1.79 (0.46; 7.04)	0.405	0.15	0.12 (0.01; 1.39)	0.089
PFUnA	0.15	Ref	0.14	1.01 (0.46; 2.24)	0.981	0.12	0.59 (0.14; 2.44)	0.467
PFHxS	1.21	Ref	1.12	0.47 (0.11; 1.95)	0.300	0.89	0.16 (0.01; 1.81)	0.138
PFOS	3.3	Ref	3.1	0.97 (0.30; 3.18)	0.963	2.35	0.49 (0.05; 4.40)	0.523
sumPFCA	3.96	Ref	4.04	0.83 (0.12; 5.74)	0.854	3.29	0.05 (0.00; 1.48)	0.084
sumPFSA	4.82	Ref	4.84	0.87 (0.20; 3.82)	0.849	4.58	0.33 (0.02; 5.48)	0.440

[b] Concentrations of PFAAs (ng/ml) were log-transformed for the multinomial logistic regression analysis that was adjusted only for age.
