# Supplementary material for: Persistent organic pollutants and non-alcoholic fatty liver disease in morbidly obese patients: a cohort study
Source: Environ Health. 2015 Sep 29;14:79. doi: 10.1186/s12940-015-0066-z (PMC4588245; doi:10.1186/s12940-015-0066-z)
Supplement: Additional file 3: Table S3. — Associations between ALT and POPs at baseline and at 12 months. (PDF 90 kb) [file 12940_2015_66_MOESM3_ESM.pdf]

Table S3. Associations between ALT and POPs at baseline and at 12 months [a].

| | Baseline (n=115) | | 12 months (n=92) | |
|---|---|---|---|---|
| Compound | B (95% CI) | p-value | B (95% CI) | p-value |
| HCB | -0.17 (-0.38 ,0.04) | 0.109 | 0.10 (-0.10 ,0.29) | 0.328 |
| beta-HCH | -0.17 (-0.34 ,0.00) | 0.046 | 0.14 (-0.07 ,0.34) | 0.193 |
| Trans-nonachlor | -0.05 (-0.20 ,0.11) | 0.549 | 0.17 (0.02 ,0.32) | 0.032 |
| p,p'-DDE | -0.04 (-0.16 ,0.08) | 0.545 | 0.12 (-0.01 ,0.25) | 0.075 |
| PCB 118 | -0.06 (-0.23 ,0.11) | 0.484 | 0.15 (-0.03 ,0.33) | 0.092 |
| PCB 153 | -0.11 (-0.32 ,0.10) | 0.317 | 0.18 (-0.01 ,0.38) | 0.069 |
| PCB 138 | -0.11 (-0.29 ,0.07) | 0.236 | 0.15 (-0.03 ,0.34) | 0.107 |
| PCB 156 | -0.09 (-0.29 ,0.12) | 0.399 | 0.18 (-0.01 ,0.38) | 0.059 |
| PCB 180 | -0.11 (-0.37 ,0.15) | 0.404 | 0.19 (-0.02 ,0.39) | 0.081 |
| PCB 170 | -0.13 (-0.40 ,0.15) | 0.362 | 0.19 (-0.03 ,0.42) | 0.091 |
| BDE 47 | 0.11 (-0.03 ,0.26) | 0.122 | 0.05 (-0.09 ,0.19) | 0.459 |
| BDE 153 | 0.14 (0.02 ,0.27) | 0.024 | 0.15 (0.03 ,0.26) | 0.013 |
| BDE 209 | 0.00 (-0.10 ,0.09) | 0.960 | 0.02 (-0.08 ,0.12) | 0.698 |
| Sum of 6 PCBs | -0.12 (-0.34 ,0.11) | 0.302 | 0.18 (-0.02 ,0.39) | 0.078 |
| Sum of 4 BDEs | 0.05 (-0.07 ,0.16) | 0.454 | 0.07 (-0.05 ,0.20) | 0.249 |

[a] Concentrations of POPs (ng/g lipids) and ALT were log-transformed for the linear regression analysis that at baseline was adjusted only for age.
