# Supplementary material for: Persistent organic pollutants and non-alcoholic fatty liver disease in morbidly obese patients: a cohort study
Source: Environ Health. 2015 Sep 29;14:79. doi: 10.1186/s12940-015-0066-z (PMC4588245; doi:10.1186/s12940-015-0066-z)
Supplement: Additional file 4: Table S4. — Associations of ALT with POPs at baseline and at 12 months stratified by sex. (PDF 165 kb) [file 12940_2015_66_MOESM4_ESM.pdf]

Table S4. Associations of ALT with POPs at baseline and at 12 months stratified by sex.

Sex	Male				Female			
	Baseline (n=36) [a]		12 months (n=30) [b]		Baseline (n=79) [a]		12 months (n=61) [b]	
Compound	B (95% CI)	p-value	B (95% CI)	p-value	B (95% CI)	p-value	B (95% CI)	p-value
HCB	-0.42 (-0.82 ,-0.02)	0.041	0.03 (-0.35 ,0.41)	0.863	-0.14 (-0.38 ,0.11)	0.279	0.13 (-0.08 ,0.34)	0.213
β-HCH	-0.49 (-0.78 ,-0.19)	0.002	0.03 (-0.37 ,0.43)	0.885	-0.09 (-0.29 ,0.11)	0.364	0.30 (0.07 ,0.53)	0.012
Trans-nonachlor	-0.23 (-0.50 ,0.05)	0.098	0.08 (-0.25 ,0.41)	0.624	-0.07 (-0.26 ,0.12)	0.483	0.20 (0.04 ,0.36)	0.014
p,p'-DDE	-0.03 (-0.25 ,0.19)	0.784	0.28 (0.02 ,0.55)	0.038	-0.04 (-0.19 ,0.10)	0.567	0.07 (-0.07 ,0.21)	0.315
PCB 118	-0.16 (-0.44 ,0.12)	0.240	0.18 (-0.15 ,0.51)	0.273	-0.05 (-0.25 ,0.16)	0.667	0.24 (0.04 ,0.45)	0.019
PCB 153	-0.29 (-0.66 ,0.08)	0.116	0.26 (-0.16 ,0.69)	0.216	-0.16 (-0.43 ,0.10)	0.220	0.21 (-0.02 ,0.43)	0.066
PCB 138	-0.21 (-0.49 ,0.08)	0.146	0.22 (-0.19 ,0.63)	0.278	-0.14 (-0.36 ,0.09)	0.226	0.18 (-0.03 ,0.39)	0.092
PCB 156	-0.26 (-0.64 ,0.13)	0.181	0.19 (-0.19 ,0.56)	0.316	-0.16 (-0.41 ,0.09)	0.212	0.24 (0.02 ,0.45)	0.034
PCB 180	-0.38 (-0.90 ,0.14)	0.150	0.30 (-0.14 ,0.74)	0.166	-0.26 (-0.59 ,0.06)	0.108	0.23 (-0.02 ,0.47)	0.068
PCB 170	-0.41 (-0.93 ,0.12)	0.124	0.29 (-0.15 ,0.73)	0.187	-0.29 (-0.63 ,0.06)	0.100	0.22 (-0.05 ,0.48)	0.105
BDE 47	0.07 (-0.20 ,0.35)	0.582	-0.09 (-0.47 ,0.30)	0.647	0.09 (-0.09 ,0.26)	0.333	0.16 (0.03 ,0.29)	0.021
BDE 153	0.17 (-0.09 ,0.43)	0.187	0.05 (-0.27 ,0.37)	0.745	0.10 (-0.05 ,0.25)	0.178	0.18 (0.07 ,0.29)	0.002
BDE 209	0.01 (-0.14 ,0.15)	0.933	-0.03 (-0.21 ,0.16)	0.746	-0.03 (-0.15 ,0.09)	0.638	0.05 (-0.06 ,0.16)	0.345
Sum of 6 PCBs	-0.34 (-0.75 ,0.07)	0.102	0.27 (-0.17 ,0.70)	0.219	-0.19 (-0.47 ,0.09)	0.180	0.23 (-0.01 ,0.46)	0.059
Sum of 4 BDEs	0.07 (-0.13 ,0.27)	0.457	-0.07 (-0.34 ,0.21)	0.626	0.00 (-0.15 ,0.15)	0.996	0.16 (0.03 ,0.29)	0.016

[a] At baseline concentrations of POPs (ng/g lipids) and ALT were log-transformed for the linear regression analysis that was adjusted for age, BMI and fasting insulin.

[b] At 12 months concentrations of POPs (ng/g lipids) and ALT were log-transformed for the linear regression analysis that was adjusted for age, weight change (kg) and fasting insulin.
